# Study on screening of fermentation agents and optimization of the fermentation process for pharyngitis tablet residue

**DOI:** 10.3389/fvets.2022.981388

**Published:** 2022-09-15

**Authors:** Qian Liu, Wei Zhong, Xue Yang, Xiaocheng Li, Zhuang Song, Ying Meng, Hanlu Liu, Li Guo, Ting Zhang

**Affiliations:** ^1^Institute of Special Animal and Plant Sciences of Chinese Academy of Agricultural Sciences, Changchun, China; ^2^Animal Science and Technology College, Jilin Agricultural Science and Technology University, Jilin, China; ^3^Jilin Dabei Agriculture and Animal Husbandry Technology Co. Ltd., Changchun, China; ^4^Agricultural College, Chifeng University, Chifeng, China

**Keywords:** Chinese medicine residue, biological fermentation, process optimization, nutritional assessment, resource utilization

## Abstract

This study aimed to screen an appropriate starter and determine the optimal fermentation process parameters to optimize the fermentation process of nutrient components and bioactive contents in pharyngitis tablet residue. This study included two experiments. In experiment I, single-factor experimental design was used to study the effects of different biological starters (cellulase preparation; *Lactobacillus Plantarum* and *Bacillus subtilis* preparation; mixture of cellulase + *Lactobacillus Plantarum* and *Bacillus subtilis*) on the nutritional values and bioactive ingredient contents in pharyngitis tablet residue. In experiment II, orthogonal design experiment was adopted to study the effects of initial water content (45, 50, and 55%), fermentation temperature (35, 37, and 40°C), and fermentation time (24, 48, and 72 h) on the changes in nutrient components, biologically activity contents, and toxin contents of the residue after optimal fermentation agent treatment. Cellulase preparation was found to be the optimal starter. The optimal fermentation conditions were: initial water content, 55%; fermentation temperature, 37°C; and fermentation time, 72 h. The contents of aflatoxin B1 and vomit toxin were in line with Chinese feed hygiene production standards. The fermentation quality of pharyngitis tablet residue can be improved by using the optimal starter and fermentation conditions.

## Introduction

Recent years have witnessed rapid advances and upscaling in the traditional Chinese medicine industry. As natural products, Chinese medicinal materials and their extracts have broad development prospects. The optimal usage of residues after extraction of active ingredients has been an area of active research. The estimated total annual output of pharmaceutical residues in China is approximately 30 million tons (1). The residues are often subjected to simple and crude methods such as field stacking, incineration, and landfill, which may cause severe water, air, and soil pollution (2). In addition, owing to the limitations of the processing purposes, production methods, and technological constraints, the residues also contain a variety of nutrients and active ingredients, such as cellulose, hemicellulose, crude protein, polysaccharides, and alkaloids (3–5). Lack of appropriate utilization of the residues can cause a huge waste of traditional Chinese medicine resources.

Chinese medicine residue is a kind of natural feed for animals. However, its usage without any treatment is liable to affect the feeding, digestion, and utilization by animals because of its strong medicinal taste, high fiber content, poor palatability, and other characteristics. Many studies have shown that biological fermentation of Chinese pharmaceutical residues can improve its resource utilization value, increase efficiency, reduce toxicity, and save drug sources. Most of the contemporary studies on this subject have focused on the changes in the active components and the pharmacological effects of the metabolites and their application in animal production after fermentation of traditional Chinese medicine residue. However, there is a paucity of studies to determine the optimal starter for fermentation of single medicinal residue and the optimal fermentation parameters.

Therefore, in this study, we conducted biological fermentation of pharyngitis tablet residue. We determined the optimal starter and the optimal fermentation parameters with respect to three aspects, i.e., the initial water content, fermentation temperature, and fermentation time. Our findings may provide a reference for the secondary utilization of pharyngitis tablet residue.

## Materials and methods

### Experiment materials

Dried pharyngitis tablet residues after extraction were provided by Jilin Tonghua Modification Pharmaceutical Co. Ltd.

*Lactobacillus Plantarum* and *Bacillus subtilis* were used as fermentation strains. These were sourced from laboratory-preserved strains. The total number of viable bacteria was ≥5 × 10^8^ CFU/mL.

Fermentation enzyme preparation is a cellulase preparation which was purchased from Guangzhou Boshi'ao Biological and Biochemical Co. Ltd. (enzyme activity: 20000 U/g).

### Experiment design and sample collection

#### Experiment I

##### Single-factor experimental design

In this experiment, pharyngitis tablet residue was used as fermentation substrate, while corn husk, soybean meal, and corn flour were used as fermentation excipients. The initial water content was set at 50%. The single factor completely random design was adopted and four treatment groups were formed, including the control group (without any biological starter), enzyme group (0.5% cellulase), bacterial group (3 mL *Lactobacillus Plantarum* + 3 mL *Bacillus subtilis*), and enzyme + bacterial group (mixture of 0.5% cellulase + 3 mL *Lactobacillus Plantarum* + 3 mL *Bacillus subtilis*). After evenly stirring the samples, they were put into a sealed fermentation bag with a breathing valve and placed in a 37°C incubator for continuous fermentation for 72 h. There were 3 replicates per sample. After 72 h, the fermentation residue was mixed and sampled using the quartering method to determine the pH, nutrients, and various active ingredients.

#### Experiment II

##### Orthogonal experimental design

In this experiment, the optimal treatment group in experiment I was taken as the experimental group. The orthogonal test with three factors and three levels was designed. The initial water content of fermentation materials (A), fermentation temperature (B), and fermentation time (C) were taken as the experimental factors. The specific level parameters of each factor are shown in Table 1.

**Table 1:** Level table of optimization combination factors of fermentation conditions.

**Treatments**	**Initial moisture content (A)**	**Fermentation temperature (B)**	**Fermentation time (C)**
1	50% (2)	40°C (3)	24 h (1)
2	55% (3)	40°C (3)	48 h (2)
3	45% (1)	40°C (3)	72 h (3)
4	45% (1)	35°C (1)	24 h (1)
5	50% (2)	35°C (1)	48 h (2)
6	55% (3)	35°C (1)	72 h (3)
7	55% (3)	37°C (2)	24 h (1)
8	45% (1)	37°C (2)	48 h (2)
9	50% (2)	37°C (2)	72 h (3)

(1), (2) and (3) respectively represent the horizontal ordering under different factors.

The fermentation steps were as follows: the raw materials were crushed to 60 measures, and water and fermentation preparations were added to the raw materials in turn and mixed evenly according to the water content of the raw materials. Each group had 3 replicates, and fermentation culture was conducted under different conditions. At the end of fermentation, samples were taken by the quartering method for laboratory analysis.

### Chemical analyses

Ten grams of Chinese medicine residue was placed in a conical flask (capacity: 250 mL), and 90 mL distilled water was added and mixed well; the mixture was filtered with gauze and the filtrate was placed in a beaker for pH measurement using pH meter (PhS-3C, No.02220128).

The content of dry matter (DM) in feed was determined by the thermostatic drying method according to GB/T6435-2006. Crude protein (CP) content was determined by the Kjeldahl nitrogen determination method according to GB/T6432-1994. Ether Extract (EE) content was determined by the Soxhlet extraction method according to GB/T 6433-2006. The content of crude fiber (CF) was determined by the filter bag method according to GB/T6434-2006. The content of neutral detergent fiber (NDF) was determined by the filter bag method according to GB/T 20806-2006, and the content of acid detergent fiber (ADF) was determined by the filter bag method according to NY/T 1459-2007. Ash content was determined by the burning method according to GB/T6438-1992.

### Determination of bioactive ingredients

The contents of reducing sugar and flavonoid were determined by the micro method and visible spectrophotometry, respectively, using kits purchased from Beijing Solaibao Technology Co. Ltd., as per the manufacturer's instructions.

The alkaloid and lactic acid content were determined by spectrophotometry and micro method, respectively, using kits purchased from Jiangsu Keming Biotechnology Co. Ltd., as per the manufacturer's instructions.

### Determination of toxin content

Both the contents of emetic toxin and aflatoxin B1 were determined by a double-antibody one-step sandwich enzyme-linked immunosorbent assay (ELISA) kit purchased from Shanghai Optimization Biotechnology Co. Ltd.

### Statistical analysis

SAS 9.4 software was used for statistical analysis. One-way ANOVA was used for the single-factor test, and Duncan's method was used for multiple comparisons. Range analysis and a general linear model were used to analyze the variance of orthogonal test results. Data were expressed as mean ± standard deviation. *P-*values < 0.05 were considered indicative of statistical significance; *P-*values < 0.01 were considered indicative of extremely significant difference.

## Results

### Changes in nutrient contents of pharmaceutical residues after treatment with different biological starters

The DM content and the Ash content in enzyme + bacterial group were significantly higher than those in the other three groups (*P* < 0.01, Table 2), whereas the other three groups showed no significant difference in this respect (*P* > 0.05). The pH values in the control group and enzyme group were significantly higher than those in the bacterial group and enzyme + bacterial group (*P* < 0.01). There was no significant difference in this respect between the control group and enzyme group, and between the bacterial group and enzyme + bacterial group (*P* > 0.05). The CF content in bacterial group was significantly higher than that in the other three groups (*P* < 0.01); however, there was no significant difference between the other three groups in this respect (*P* > 0.01). The NDF content in bacterial group was significantly higher than that in the enzyme group and enzyme + bacterial group (*P* < 0.01), but not significantly different from that in the control group (*P* > 0.05); however, the NDF content in the enzyme group was significantly lower than that in the control group (*P* < 0.01). The ADF content in the bacterial group was significantly higher than that in the control group and enzyme group (*P* < 0.05), whereas there was no significant difference between bacterial group and enzyme + bacterial group or between enzyme group and control group in this respect (*P* > 0.05). The EE content and CP content were not significantly affected by different starter treatments (*P* > 0.05).

**Table 2:** Changes in nutrient contents of pharmaceutical residues after treatment with biological starter.

**Items**	**Control group**	**Enzyme group**	**Bacterial group**	**Enzyme + bacterial group**	***P*-value**
DM (%)	43.94 ± 0.24^B^	44.13 ± 0.91^B^	43.98 ± 0.18^B^	45.41 ± 0.94^A^	<0.0001
pH-value	4.13 ± 0.01^A^	4.14 ± 0.00^A^	4.06 ± 0.01^B^	4.05 ± 0.00^B^	<0.0001
EE (%)	2.94 ± 0.25	3.06 ± 0.15	2.91 ± 0.53	3.08 ± 0.29	0.6222
CP (%)	13.83 ± 0.53	12.33 ± 3.51	13.74 ± 0.77	13.47 ± 0.27	0.3077
CF (%)	20.31 ± 0.71^B^	20.19 ± 0.94^B^	22.48 ± 0.63^A^	20.69 ± 1.40^B^	0.0002
NDF (%)	47.48 ± 2.26^AB^	43.34 ± 0.99^C^	49.22 ± 1.68^A^	45.16 ± 2.36^BC^	<0.0001
ADF (%)	32.27 ± 1.73^b^	31.74 ± 1.30^b^	33.62 ± 0.90^a^	32.71 ± 1.22^ab^	0.0344
Ash (%)	12.42 ± 0.12^B^	12.29 ± 0.19^B^	12.48 ± 0.32^B^	12.83 ± 0.30^A^	0.0005

Different capital letters of shoulder labels in the same data indicate an extremely significant difference (P < 0.01), different lowercase letters indicate a significant difference (P < 0.05), and no letters indicate an insignificant difference (P > 0.05). The same as below.

### Changes of bioactive ingredients in residues treated by different biological starters

The flavonoid content in the enzyme group was significantly higher than that in the other three groups (*P* < 0.01, Table 3), while there was no significant difference between the other three groups in this respect (*P* > 0.05). The reducing sugar content in enzyme group was significantly higher than those in the other three groups (*P* < 0.01), and that in the enzyme + bacterial group was significantly higher than that in control group and bacterial group (*P* < 0.01); however, there was no significant difference between the control group and bacterial group in this respect (*P* > 0.05). The different biological starters had no significant effect on the alkaloid and lactic acid content in residue after fermentation among different groups (*P* > 0.05).

**Table 3:** Content changes of bioactive components in pharmaceutical residues after treatment with biological starters.

**Items**	**Control group**	**Enzyme group**	**Bacterial group**	**Enzyme + bacterial group**	***P*-value**
Flavonoid (mg/g)	2.91 ± 0.26^B^	3.92 ± 0.28^A^	2.81 ± 0.29^B^	2.45 ± 0.18^B^	0.0006
Reducing sugar (μg/g)	4.68 ± 0.04^C^	9.43 ± 0.31^A^	4.89 ± 0.05^C^	7.94 ± 0.21^B^	<.0001
Alkaloid (μg/g)	4.66 ± 2.09	1.52 ± 0.15	3.61 ± 1.06	2.57 ± 0.92	0.0726
Lactic acid (μmol/g)	308.41 ± 10.17	303.69 ± 7.26	325.63 ± 10.46	310.57 ± 5.72	0.0648

### Change of DM, pH, EE, CP, Ash contents in residues under different fermentation conditions

The primary and secondary order of factors affecting DM content was A>C>B (Table 4). For improving DM content, the optimal combination was A_1_B_1_C_2._ The results showed that the initial water content was 45%, the fermentation temperature was 35°C, and the fermentation time was 48 h. Initial water content and fermentation time showed extremely significant effect on DM content (*P* < 0.01, Table 5), while fermentation temperature had significant effect on DM content (*P* < 0.05).

**Table 4:** Analysis table of nutrient range of Chinese medicine residue after fermentation.

**Treatments**		**Factors**			**Indices**				
		**A**	**B**	**C**	**DM**	**pH**	**EE**	**CP**	**Ash**
1		2	3	1	56.93	4.63	2.50	12.10	10.50
2		3	3	2	51.14	4.27	2.66	12.08	11.46
3		1	3	3	61.21	4.51	2.47	11.86	11.75
4		1	1	1	61.63	4.72	2.67	12.12	12.62
5		2	1	2	56.63	4.28	2.66	12.08	12.21
6		3	1	3	51.26	4.06	2.77	12.80	13.59
7		3	2	1	50.65	4.23	2.78	12.78	12.01
8		1	2	2	62.06	4.53	2.59	13.33	12.44
9		2	2	3	55.87	4.15	3.06	13.44	12.29
DM	K1	61.63	56.51	56.40				
	K2	56.48	56.19	56.61	Row rank A > C > B				
	K3	51.02	56.43	56.11	A_1_B_1_C_2_				
	Range	10.61	0.32	0.50				
pH	K1	4.59	4.35	4.53				
	K2	4.35	4,30	4.36	Row rank A > C > B				
	K3	4.19	4.47	4.24	A_3_B_2_C_3_				
	Range	0.40	0.17	0.29				
EE	K1	2.58	2.70	2.65				
	K2	2.74	2.81	2.64	Row rank B > A > C				
	K3	2.74	2.54	2.77	A_2_B_2_C_3_				
	Range	0.16	0.27	0.13				
CP	K1	12.44	12.33	12.33				
	K2	12.54	13.18	12.50	Row rank B > C > A				
	K3	12.55	12.01	12.70	A_3_B_2_C_3_				
	Range	0.11	1.17	0.37				
Ash	K1	12.27	12.81	11.71				
	K2	11.67	12.25	12.04	Row rank B > C > A				
	K3	12.35	11.24	12.54	A_2_B_3_C_1_				
	Range	0.68	1.57	0.83				

**Table 5:** Analysis of variance of nutritional components of Chinese medicinal residues after fermentation.

**Indices**	**Source**	**df**	**Sum of squares**	**Mean square**	***F*-value**	**Pr > F**
DM	Model	6	1,504	250.7	606.23	<0.0001
	A	2	1,495	747.4	1808**	<0.0001
	B	2	2.67	1.34	3.23*	0.0453
	C	2	6.56	3.28	7.93**	0.0008
	Pure error	73	30.18	0.41		
	Cor total	79	1534			
pH	Model	6	3.70	0.62	433.05	<0.0001
	A	2	2.18	1.09	765.58**	<0.0001
	B	2	0.38	0.19	134.44**	<0.0001
	C	2	1.14	0.57	399.13**	<0.0001
	Pure error	74	0.11	0.0014		
	Cor total	80	3.81			
EE	Model	6	1.71	0.28	5.71	<0.0001
	A	2	0.46	0.23	4.62*	0.0129
	B	2	0.97	0.49	9.75**	0.0002
	C	2	0.27	0.14	2.74	0.0710
	Pure error	73	3.69	0.05		
	Cor total	79	5.40			
CP	Model	6	21.66	3.61	9.60	<0.0001
	A	2	0.22	0.11	0.29	0.7487
	B	2	19.63	9.82	26.11**	<0.0001
	C	2	1.81	0.91	2.41	0.0970
	Pure error	74	27.81	0.38		
	Cor total	80	49.47			
Ash	Model	6	51.27	8.54	31.64	<0.0001
	A	2	7.56	3.78	14.00**	<0.0001
	B	2	34.22	17.11	63.37**	<0.0001
	C	2	9.48	4.74	17.56**	<0.0001
	Pure error	74	19.98	0.27		
	Cor total	80	71.25			

^*^Significant difference, P < 0.05; ^**^was significantly different, P < 0.01. The same as below.

The primary and secondary order of factors affecting pH value was A>C>B (Table 4). For improving pH value, the optimal combination was A_3_B_2_C_3._ The results showed that the initial water content was 55%, the fermentation temperature was 37°C, and the fermentation time was 72 h. The initial water content, fermentation temperature, and fermentation time had extremely significant effect on the pH value (*P* < 0.01, Table 5).

The primary and secondary order of factors affecting EE content was B>A>C (Table 4). For improving EE content, the best combination was A_2_B_2_C_3._ The results showed that the initial water content was 50%, the fermentation temperature was 37°C, and the fermentation time was 72 h. The initial water content showed a significant effect on this index (*P* < 0.05, Table 5), the fermentation temperature showed a very significant effect on EE content (*P* < 0.01), while the fermentation time had no significant effect on EE content (*P* > 0.05).

The primary and secondary order of factors affecting CP content was B>C>A (Table 4). For improving CP content, the optimal combination was A_3_B_2_C_3_. The results showed that the initial water content was 55%, the fermentation temperature was 37°C, and the fermentation time was 72 h. Fermentation temperature had extremely significant effect on CP content (*P* < 0.01, Table 5), while the initial water content and fermentation time had no significant effect on CP content (*P* > 0.05).

The primary and secondary order of factors affecting Ash content was B>C>A (Table 4). For Ash content, the best combination was A_2_B_3_C_1_._._The results showed that the initial water content was 50%, the fermentation temperature was 40°C, and the fermentation time was 24 h. Initial water content, fermentation temperature, and fermentation time all had extremely significant effect on this index (*P* < 0.01, Table 5).

### Changes of CF, NDF, ADF contents in residues under different fermentation conditions

The primary and secondary order of factors affecting CF content was B>A>C (Table 6). For reducing CF content, the optimal combination was A_3_B_3_C_3._ The results showed that the initial water content was 55%, fermentation temperature was 40°C, and the fermentation time was 72 h. The initial water content, fermentation temperature, and fermentation time all showed an extremely significant effect on CF content (*P* < 0.01, Table 7).

**Table 6:** Analysis table of different fiber content range after Chinese medicine residue fermentation.

**Treatments**		**Factors**			**Indices**		
		**A**	**B**	**C**	**CF**	**NDF**	**ADF**
1		2	3	1	18.37	44.21	30.85
2		3	3	2	16.87	41.26	29.69
3		1	3	3	18.35	44.21	31.33
4		1	1	1	19.43	47.19	32.70
5		2	1	2	19.26	45.52	32.38
6		3	1	3	16.65	43.69	32.37
7		3	2	1	20.33	43.74	31.56
8		1	2	2	21.31	46.75	33.53
9		2	2	3	19.66	43.34	31.74
CF	K1	19.70	18.45	19.38		
	K2	19.10	20.43	19.15	Row rank B > A > C		
	K3	17.95	17.86	18.22	A_3_B_3_C_3_		
	Range	1.75	2.57	1.16		
NDF	K1	46.05	45.47	45.05		
	K2	44.36	44.61	44.51	Row rank A > B > C		
	K3	42.90	43.23	43.75	A_3_B_3_C_3_		
	Range	3.15	2.24	1.30		
ADF	K1	32.52	32.48	31.70		
	K2	31.66	32.28	31.87	Row rank B > A > C		
	K3	31.21	30.62	31.81	A_3_B_3_C_1_		
	Range	1.31	1.86	0.17		

**Table 7:** Analysis of variance of various fiber contents after fermentation of Chinese medicinal residue.

**Indices**	**Source**	**df**	**Sum of squares**	**Mean square**	***F*-value**	**Pr > F**
CF	Model	6	160.45	26.74	14.30	<.0001
	A	2	42.36	21.18	11.33**	<.0001
	B	2	97.93	48.96	26.19**	<.0001
	C	2	20.17	10.09	5.39**	0.0065
	Pure error	74	138.36	1.87		
	Cor total	80	298.82			
NDF	Model	6	226.84	37.81	11.57	<.0001
	A	2	134.67	67.33	20.60**	<.0001
	B	2	69.02	34.51	10.56**	<.0001
	C	2	23.15	11.57	3.54*	0.0340
	Pure error	74	241.88	3.27		
	Cor total	80	468.72			
ADF	Model	6	80.15	13.36	5.03	0.0002
	A	2	23.95	11.97	4.51*	0.0142
	B	2	55.84	27.92	10.52**	<.0001
	C	2	0.36	0.18	0.07	0.9337
	Pure error	74	196.34	2.65		
	Cor total	80	276.50			

The primary and secondary order of factors affecting NDF content was A>B>C (Table 6). For reducing NDF content, the best combination was A_3_B_3_C_3_, The results showed that the initial water content was 55%, fermentation temperature was 40°C, and the fermentation time was 72 h. The initial water content and fermentation temperature had extremely significant effect on this index (*P* < 0.01, Table 7), and the fermentation time had significant effect on this index (*P* < 0.05).

The primary and secondary order of factors affecting ADF content was B>A>C (Table 6). For reducing ADF content, the optimal combination was A_3_B_3_C_1_. The initial water content was 55%, the fermentation temperature was 40°C, and the fermentation time was 24 h. The initial water content and the fermentation temperature had a significant effect on ADF content (*P* < 0.01, Table 7). However, the fermentation time had no significant effect on ADF content (*P* > 0.05).

### Changes in biologically activity contents in residues under different fermentation conditions

The primary and secondary order of factors affecting flavonoid content was A>B>C (Table 8). For flavonoid content, the optimal combination was A_3_B_2_C_1_, wherein the initial water content was 55%, the fermentation temperature was 37°C, and the fermentation time was 24 h. The initial water content, fermentation temperature, and fermentation time had no significant influence on flavonoid content (*P* > 0.05, Table 9).

**Table 8:** Analysis table of range of bioactive components in Chinese medicine residue after fermentation.

**Treatments**	**Factors**			**Indices**			
	**A**	**B**	**C**	**Flavonoid**	**Reducing sugar**	**Alkaloid**	**Lactic acid**
1		2	3	1	2.23	6.59	7.75	189.70
2		3	3	2	1.02	5.49	10.89	318.24
3		1	3	3	0.08	7.77	9.63	243.15
4		1	1	1	0.41	5.47	6.18	160.83
5		2	1	2	1.69	5.14	8.36	279.24
6		3	1	3	3.35	5.41	5.51	323.48
7		3	2	1	3.92	6.03	2.35	260.20
8		1	2	2	1.67	5.23	2.48	232.21
9		2	2	3	1.20	9.43	1.52	302.56
Flavonoid	K1	0.72	1.82	2.18			
	K2	1.71	2.26	1.46	Row rank A > B > C			
	K3	2.76	1.11	1.54	A_3_B_2_C_1_			
	Range	2.04	1.15	0.73		
Reducing sugar	K1	6.16	5.34	6.03			
	K2	7.05	6.90	5.29	Row rank C > B > A			
	K3	5.64	6.62	7.54	A_2_B_2_C_3_			
	Range	1.41	1.56	2.25			
Alkaloid	K1	6.10	6.68	5.43			
	K2	5.88	2.12	7.24	Row rank B > C > A			
	K3	6.25	9.42	5.55	A_3_B_3_C_2_			
	Range	0.37	7.30	1.81			
Lactic acid	K1	212.06	254.52	203.58	Row rank A > C > B			
	K2	257.17	264.99	276.56			
	K3	300.64	250.36	289.73	A_3_B_2_C_3_			
	Range	88.58	14.63	86.15			

**Table 9:** Analysis of variance of bioactive components of Chinese medicinal residues after fermentation.

**Indices**	**Source**	**df**	**Sum of squares**	**Mean square**	***F*-value**	**Pr > F**
Flavonoid	Model	6	9.22	1.54	0.84	0.6348
	A	2	6.25	3.12	1.70	0.3705
	B	2	2.02	1.01	0.55	0.6451
	C	2	0.95	0.47	0.26	0.7956
	Pure error	2	3.68	1.84		
	Cor total	8	12.90			
Reducing sugar	Model	6	15.09	2.51	3.03	0.2685
	A	2	3.06	1.53	1.84	0.3517
	B	2	4.15	2.07	2.50	0.2855
	C	2	7.88	3.94	4.76	0.1737
	Pure error	2	1.66	0.83		
	Cor total	8	16.74			
Alkaloid	Model	6	88.11	14.69	8.19	0.1128
	A	2	0.21	0.10	0.07	0.9449
	B	2	81.76	40.88	22.80*	0.0420
	C	2	6.14	33.07	1.71	0.3686
	Pure error	2	3.59	1.79		
	Cor Total	8	91.70			
Lactic acid	Model	6	25,033	4,172	27.83	0.0351
	A	2	11,769	5,885	39.25*	0.0248
	B	2	340.93	170.46	1.14	0.4680
	C	2	12,923	6,462	43.10*	0.0227
	Pure error	2	299.87	149.93		
	Cor total	8	25,333			

The primary and secondary order of factors affecting reducing sugar content was C>B>A (Table 8). To increase the reducing sugar content, the best combination was A_3_B_3_C_2_, wherein the initial water content was 50%, the fermentation temperature was 37°C, and the fermentation time was 72 h. The initial water content, fermentation temperature, and fermentation time had no significant influence on reducing sugar content (*P* > 0.05, Table 9).

The primary and secondary order of factors affecting alkaloid content was B>C>A (Table 8). For alkaloid content, the best combination was A_3_B_1_C_2_, wherein the initial water content was 55%, the fermentation temperature was 40°C, and the fermentation time was 48 h. Fermentation temperature had a significant effect on alkaloid content (*P* < 0.05, Table 9), while the initial water content and fermentation time had no significant effect on this index (*P* > 0.05).

The primary and secondary order of factors affecting lactic acid content was A>C>B (Table 8). For increasing lactic acid content, the best combination was A_3_B_2_C_3_, wherein the initial water content was 55%, the fermentation temperature was 37°C, and the fermentation time was 72 h. Initial water content and fermentation time had significant effect on this index (*P* < 0.05, Table 9), whereas fermentation temperature had no significant effect on this index (*P* > 0.05).

### Determination of toxin contents under different fermentation conditions

The contents of aflatoxin B1 and vomit toxin in fermented pharmaceutical residues were in line with the Chinese feed hygiene production standards (Table 10).

**Table 10:** Toxin contents of pharyngitis tablet residue after fermentation.

**Treatments**	**AFB1 (ppb)**	**Testing results (≤ 20ppb)**	**DON (ppb)**	**Testing results (≤ 1,000ppb)**
1	1.48	Qualified	231.90	Qualified
2	1.12	Qualified	206.90	Qualified
3	1.11	Qualified	216.90	Qualified
4	1.38	Qualified	211.90	Qualified
5	1.06	Qualified	215.90	Qualified
6	1.05	Qualified	184.90	Qualified
7	1.05	Qualified	222.90	Qualified
8	0.92	Qualified	306.90	Qualified
9	0.96	Qualified	223.90	Qualified

## Discussion

### Effects of different starter treatments on fermentation effect of pharyngitis tablet residue

Lactic acid bacteria, Bacillus and cellulase are commonly used as feed fermentation additives. The starters can directly or indirectly alter the physical or chemical structure of feed raw materials (6, 37), such as reducing the fiber content and antinutritional factors of the feed (7, 40), and improving palatability, nutritional value, and utilization efficiency. The results showed that the CF, NDF, and ADF contents in the enzyme group were 0.59, 8.72, and 1.64% lower (respectively) than those in the control group. The CF, NDF, and ADF contents in the enzyme group were 10.19, 13.56, and 5.59% lower than those in bacterial group and were 2.42, 4.03, and 2.97% lower than those in the enzyme + bacterial group, respectively. This is consistent with the research results of Ye (8) and Wang (9) indicating that enzyme fermentation can improve the nutritional composition and quality of pharmaceutical residues. The CF, NDF, and ADF contents in the bacterial group were significantly higher than those in the control group and other groups. This was different from the results reported by Su et al. (10) and Ahmed et al., (43) which may be related to the limited ability of probiotic lactic acid bacteria and bacillus to degrade fiber. Compared with single strain fermentation, mixed strain fermentation offers advantages due to the comprehensive effect of bacteria and enzymes, improving the fermentation efficiency and fermentation quality (11). After microbial fermentation, cellulase, pectinase, and other extracellular enzymes produced by microorganisms enter the culture medium, resulting in the rupture of herbal cells and exposure of active ingredients. In this experiment, compared to the control group, enzyme group > enzyme + bacterial group > bacterial group with respect to degradation of fiber content. Enzyme + bacterial group did not show additional advantage with respect to fiber content compared to the control group and other groups. Enzyme group was superior to enzyme + bacterial group, which could be related to the strain screening and combination, or fermentation conditions.

Dry matter (DM) content influences the fermentation quality. Lower DM content is not easy to ferment, whereas higher DM can reduce palatability, which affects animal feed intake. Xu et al. (12) reported that 30-40% DM content was relatively appropriate. The DM content measured in this study was approximately 43%, and the DM content in enzyme group, bacterial group, and enzyme + bacterial group was 0.43, 0.09, and 3.35% higher than that in the control group, respectively. This indicated that the fermentation water content was well controlled and conducive to the improvement of fermentation quality (13–15). Studies have shown that biological fermentation can decrease the pH value and improve fermentation quality. In this study, the pH values of bacteria group and enzyme + bacteria group were lower than those of control group, which was consistent with previous studies. However, in the present study, the pH value in the enzyme group was higher than that in the control group. This may be attributable to retention of some residual air during the process of sealing the fermentation bags leading to the formation of competitive growth relationships among fermentation strains.

Flavonoids, reducing sugars, alkaloids, and lactic acid are important bioactivity indices to verify the quality of fermentation. In a study by Zhang et al. (16), the flavonoid content in fermentation products was found to be related to fermentation time, and the content increased with the extension of fermentation time for a certain period, and then showed a decreasing trend. Liu et al. (17) found that flavonoid content in Roxburgh rose residue after fermentation was lower than that before fermentation. In the present study, flavonoid content in the enzyme group was 34.48, 39.29, and 62.5% higher than that in the control group, bacterial group, and the enzyme + bacterial group, respectively. However, flavonoids in bacterial group and enzyme + bacterial group were not significantly different compared to the control group. The reducing sugar content in the enzyme group was 101.50, 92.8, and 18.77% higher than that in control group, bacterial group, and enzyme + bacterial group, respectively. In previous studies, enzyme preparation was added to forage primarily to induce breakdown of plant cell walls at ensiling to improve silage fermentation by providing sugars for the LAB and to enhance the nutritive value of silage by increasing the digestibility of cell walls. Cellulase offers a distinct advantage in fiber degradation (18, 19). Adding cellulase to feed can release bioactive substances through degradation for microbial metabolic activities (20, 21). The reducing sugar content in enzyme + bacterial group was 69.66 and 62.37% higher than that in control group and bacterial group, respectively. However, there was no significant difference between control group and bacterial group in this respect. This indicated that the addition of appropriate enzyme preparation had a significant positive effect on the degradation of cellulose into reducing sugar and release of flavonoids, which may be due to a certain relationship between flavonoid contents after fermentation and in the residue substrate (22). The enzyme can effectively destroy the cell wall and release the active ingredients in the residue. The ability to break the bacterial wall is limited, and the amount of enzyme produced by the enzyme + bacteria group is limited, which is not as good as the effect in the enzyme group. Alkaloids are a class of nitrogen-containing basic organic compounds commonly found in plants (11). In this experiment, fermentation had no significant effect on the alkaloid content. Possibly due to the extraction process, the amount of residue was less, which can be proved by the alkaloid content data. Studies have shown a significant negative correlation between lactic acid content and pH value (23). In this paper, there is a negative correlation between lactic acid content and pH value, which is consistent with the results of previous studies. Lactic acid is produced by lactic acid bacteria and other microorganisms in the fermented feed. It is the highest content of acid in the fermented feed. The level of lactic acid content can directly improve the fermentation effect (24, 25). In this experiment, lactic acid content in the bacteria group and enzyme + bacteria group were higher than that in the control group, which may be due to the fact that *Lactobacillus Plantarum* is more likely to start fermentation after its addition to the residue and promote the strain to generate more lactic acid. The content in the enzyme treated group was lower than that in the control group, which may be caused by the low lactic acid content generated by the degradation of cellulose into glucose by cellulase. It may also be due to the reaction of lactic acid with other substances or volatilization during the heating and drying process, which will also lead to the loss of some lactic acid.

### Effect of initial water content on fermentation effect of pharyngitis tablet residue

Initial water content affects the fermentation quality. Studies have shown that when the water content of the fermented feed is 40–60%, the pH of the feed can be maintained stable (26), and the strain activity is higher (27, 28). Both Wei et al. (29) and Chen et al. (30) demonstrated that 50% initial water content of *Astragalus membranaceus* and *Astragalus membranaceus* residues was good for fermentation. However, the composition of different residues is different, and the appropriate initial water content is liable to vary. In this study, the initial water content of 55% was good for the fermentation of pharyngitis tablet residue.

### Effect of fermentation temperature on the fermentation effect of pharyngitis tablet residue

Temperature is an important factor affecting the growth and metabolism of strains. The reported appropriate temperatures for the growth of *Lactobacillus Plantarum* and *Bacillus subtilis* were 30–37°C and 25–40°C, respectively (31, 42). The optimum temperature for cellulase is 37–50°C (39). Studies have shown that optimal temperature can facilitate more vigorous growth of strains, with higher number of viable bacteria and higher enzyme activity so as to ensure better fermentation quality. In this experiment, the optimum temperature was 37°C for the best fermentation effect of pharyngitis tablet residue. This was not consistent with the previously reported optimal fermentation temperature of 33°C (32) or 30°C (33) for medicinal residue. This may be due to the influence of fermentation substrate and fermentation strain on the optimal fermentation temperature. In the study by Jiang et al. (34), fermentation temperature of 36.6°C was found to be beneficial for the compound microbial agent to improve the silage quality of sweet sorghum residue. The results are similar to those of this experiment, which confirms the reliability of the fermentation results.

### Effect of fermentation time on the fermentation effect of pharyngitis tablet residue

Fermentation time is also an important determinant of fermentation quality. During the fermentation process, with the extension of the fermentation time, microorganisms continuously multiply by consuming nutrients, resulting in changes in the active ingredients and nutritional value of pharmaceutical residues (38). Yin et al. (35) found that the optimal fermentation time for the fermentation process of glucose generation from pine residue was 70 h. Liu (36) found that the optimal fermentation time of Aspergillus Niger H-2 for cellulase production was 72 h, and the optimal fermentation time of *Astragalus membranaceus* residue was 120 h. In the present study, the optimal fermentation time was 72 h. This was consistent with the study by Yin et al. (35) and Liu (36). In the present study, cellulase degraded cytoderm of residue and provided substrate for the growth of lactic acid bacteria so as to promote the fermentation reaction. However, Zhu et al. (41) reported an optimal fermentation time of 48 h with similar strains. This discrepancy may be attributable to different types of medical residues and the dose of added strains.

### Effect of fermentation conditions on toxin contents

Aflatoxin B1 and emetic toxin are the key indices for evaluating feed safety. Feed Production Safety Standard requires AFB1 ≤ 20 ppb and DON ≤ 1000 ppb. In this experiment, the AFB1 and DON contents of pharyngitis tablet residue after different fermentation conditions were in line with the Chinese feed safety standards. This indicates the safety of pharyngitis tablet residue after fermentation for use as animal feed in livestock and poultry production.

## Conclusion

In the experiment, the optimal fermentation starter for pharyngitis tablet residue was cellulase and the optimal fermentation conditions were: initial water content 55%; fermentation temperature 37°C; and fermentation time, 72 h. Suitable fermentation conditions can improve the nutritional value of Chinese medicine residue. The fermented Chinese medicine residue will not only solve the problem of environmental pollution but also be used as a new feed resource applied in animal production in the future.

## Data availability statement

The original contributions presented in the study are included in the article/supplementary material, further inquiries can be directed to the corresponding author.

## Author contributions

QL conceived and designed research, conducted the experiments, analyzed the data, and drafted the manuscript. WZ, HL, XY, and LG conceived and designed research and reviewed and edited the manuscript. TZ, XL, ZS, and YM provided the experimental samples and materials. All authors contributed to the article and approved the submitted version.

## Funding

This research was funded by Start-up Fund for Scientific Research of Jilin Agricultural Science and Technology University (20220031), Enterprise Project Support from Jilin Dabei Agriculture and Animal Husbandry Technology Co. Ltd., (cgc-20210506-3-4), and the Science and Technology Innovation Program of Chinese Academy of Agricultural Sciences (CAAS-ASTIP-2021-ISAPS). This study received funding from Animal Husbandry Technology Co. Ltd.. The funder was not involved in the study design, collection, analysis, interpretation of data, the writing of this article, or the decision to submit it for publication.

## Conflict of interest

Authors XL, ZS, and YM were employed by Jilin Dabei Agriculture and Animal Husbandry Technology Co. Ltd.

The remaining authors declare that the research was conducted in the absence of any commercial or financial relationships that could be construed as a potential conflict of interest.

## Publisher's note

All claims expressed in this article are solely those of the authors and do not necessarily represent those of their affiliated organizations, or those of the publisher, the editors and the reviewers. Any product that may be evaluated in this article, or claim that may be made by its manufacturer, is not guaranteed or endorsed by the publisher.
